# Stridulatory Organs and Sound Recognition of Three Species of Longhorn Beetles (Coleoptera: Cerambycidae)

**DOI:** 10.3390/insects15110849

**Published:** 2024-10-30

**Authors:** Jia-Quan Wei, Xiao-Yun Wang, Xia-Lin Zheng, Xin Tong

**Affiliations:** Guangxi Key Laboratory of Agro-Environment and Agric-Products Safety, National Demonstration Center for Experimental Plant Science Education, College of Agriculture, Guangxi University, Nanning 530004, China; 2217392049@st.gxu.edu.cn (J.-Q.W.); wxy8771@163.com (X.-Y.W.); zheng-xia-lin@163.com (X.-L.Z.)

**Keywords:** average recognition rate, insect classification, insect sounds, Lamiinae, longicorn beetle, sound-producing mechanisms

## Abstract

Sound is an important medium of communication among insects. Some longhorn beetles produce sounds during their daily activities, and these sounds play a role in courtship, predation, and defense. However, whether there are differences in the sounds emitted by longhorn beetles and how to distinguish and recognize these sounds have not been investigated in detail. Here, the sounds of *Glenea cantor* (Fabricius), *Moechotypa diphysis* (Pascoe), and *Psacothea hilaris* (Pascoe) were collected, and the differences in their stridulatory organs were observed and compared using scanning electron microscopy (SEM). The characteristics of their sounds were analyzed using MATLAB. Linear prediction cepstral coefficients (LPCC) and Mel frequency cepstral coefficients (MFCC) were used to extract the sound features, and the support vector machine (SVM) model was used to identify the sounds of three species. The results showed that the stridulatory organs of three species of longhorn beetles differed in morphology and time domain, and the combination of MFCC and SVM had a better recognition ability. The difference in the stridulatory organs of longhorn beetles may be an important reason for the differences in the sounds they produce, and we discussed the application of insect sounds in insect classification.

## Simple Summary

Longhorn beetles are agricultural and forest economic pests, and some of them can rely on the friction of the thoracic stridulatory organs to produce sounds. The stridulatory organs are composed of the file on the mesoscutum and the scraper on the pronotum. In this study, we collected the sounds of three species of Lamiinae longhorn beetles to compare their thoracic stridulatory organs, and found there were significant inter- and intraspecific variations in morphology and time domain. We also used sound recognition technology to identify and classify the sounds of these three longhorn beetles. We hope to provide a method for the identification and classification of longhorn beetles through sound recognition technology.

## 1. Introduction

Insects can obtain information about their surroundings through their sensory organs [1]. For example, male moths can rely on antennae to sense sexual information and respond [2]. Ants are able to detect danger and realize intraspecific communication with their antennae and thus are able to forage, alert conspecifics, and convey information with sound [3]. Bees communicate information through pheromones, dancing, and wing flapping [4].

Sound is one of the effective ways for insects to communicate, and plays a crucial role in insect courtship, predation, and defense [5,6,7]. For example, cicada males emit sounds that can attract the female to mate [8,9]. The sound emitted by crickets not only serves as a means of courtship, but also serves as a warning when they detect danger [10]. In male–male competitions among bark beetles, larger males can often win by emitting louder, lower sounds [11].

Different insect stridulatory organs and sound-producing mechanisms lead to the distinctive sounds produced by insects [12,13,14]. For example, the vocalizations of caterpillars can be divided into long units and short units. There are significant differences in the main frequency and relative amplitude of these two units [15]. The stridulatory organs of kissing bug *Mepraia spinolai* are different in alary morphotypes, and their spectral properties are different between the two sexes [16]. The head and chest of the longhorn beetles rub against each other to make sounds, during which the longhorn beetles make a regular nodding movement [17,18].

In recent years, entomologists have utilized the unique sounds of insects for detection, recognition, and classification [6,19,20]. For example, acoustic analysis has been used in postharvest management for monitoring rice weevils [21,22]. Towsey et al. [23] demonstrated that the use of acoustic indices could identify the cicada chorus in the natural environment. Kawakita and Ichikawa [24] conducted acoustic analysis to accurately identify bees and hornets using their flight sounds. The application of these automatic recognition methods could efficiently monitor insect activities and quickly identify insect species. These methods allow non-experts to successfully identify insects and implement specific insect management [21,22,23,24].

Longhorn beetles are wood-infesting insects that damage the phloem and xylem of plants during the larvae stage, which can cause tree death. Adults may feed on twigs, which causes tree damage and potential for introducing additional tree mortality agents such as nematodes and microbial pathogens [25,26]. The larvae of longhorn beetles can produce three types of sounds: sliding, scraping, and snapping. There are some studies dedicated to utilizing acoustic detection methods to analyze whether the larvae exist in the tree trunks, thus determining the health of trees [27,28]. This method has significant economic value. As for the adults of longhorn beetles, only three subfamilies: Lamiinae, Cerambycinae, and Lepturinae can produce sound. Their males and females rely on the mutual movement of the file and scraper to produce frictional sounds [29]. Cheng [30] observed and recorded the sound waveforms of 15 longhorn beetles and found that the sound of each species of longhorn beetle was unique. By investigating the sounds of different longhorn beetle species and developing sound recognition methods, we can possibly solve the difficult problem of species identification in the field. This is likely to help agricultural workers or insect experts to identify the common longhorn beetles quickly, and thus improve the efficiency of pest control or insect classification.

The process of sound recognition in insects is mainly divided into two steps. Firstly, it is necessary to extract the features of the sound, and commonly used extraction methods including linear prediction cepstral coefficients (LPCC) and Mel frequency cepstral coefficients (MFCC). Secondly, the recognition model must be selected; a typical method is the support vector machine (SVM) [31,32]. However, there is still no consensus on which sound feature extraction method combining with the SVM model is better for insect sound recognition.

In this paper, we analyzed and compared the stridulatory organs, morphology, and acoustic features of three common species of longhorn beetles in the Lamiinae–*Glenea cantor* (Fabricius), *Moechotypa diphysis* (Pascoe), and *Psacothea hilaris* (Pascoe)–using SEM. Extraction of acoustic features was performed by LPCC and MFCC, and recognition by SVM modeling. We analyzed and classified the sounds emitted by the three species of longhorn beetles, with the aim of providing a new approach for longhorn beetles recognition and classification.

## 2. Materials and Methods

### 2.1. Insect Collection and Rearing

*Glenea cantor* adults were collected in Qingxiushan Park, Nanning, Guangxi (108°23′ E, 22°47′ N) in May 2019, *Moechotypa diphysis* adults were collected in Kuandian County, Liaoning Province (123°22′–125°42′ E, 39°43′–41°09′ N) in October 2021, and *Psacothea hilaris* adults were collected in Fusui County, Guangxi (107°31′–108°06′ E, 22°17′–22°57′ N) in August 2022. The collected adults were reared in 40 cm × 30 cm × 30 cm cages and given branches of kapok, chestnut, and mulberry trees, respectively, at a temperature of 25 ± 2 °C, a humidity of 80 ± 5%, and a photoperiod of 14:10 h (L:D).

### 2.2. Sound Collection

In order to make the longhorn beetle produce sounds, it was necessary to pinch the elytra of the adult longhorn beetle with the index finger and thumb. Then, we used a Sony Linear PCM Recorder D-100 audio recorder (Sony, Tokyo, Japan) to aim at the junction of the head and thorax of the adults (the position of stridulatory organ) to record the sounds (1–2 cm from the longhorn beetle) with a sampling resolution of 44.1 kHz/24-bit, and the result was saved in WAV file format. For each species of longhorn beetle, 15 females and 15 males with complete bodies and active behavior were selected.

### 2.3. Preprocessing and Analysis

The collected sounds were edited and processed by Adobe Audition 2022 to obtain high-quality audio, and then analyzed for their acoustic features, including the time domain and frequency domain using MATLAB R2022b. The preprocessed sounds of the three species of longhorn beetles were divided into a training set and a test set. The training set stored the audio samples of each longhorn beetle, and the test set stored the audio samples to be tested for each longhorn beetle. The training set of each longhorn beetle contained 60 sound signals, and the test set increased from 10 to 100 sound signals. LPCC and MFCC were used to extract features from these audio samples, respectively. The recognition model used SVM and the five-fold cross-validation method.

### 2.4. Light Microscopy and SEM

The stridulatory organs of three species of longhorn beetles were observed by SEM. The file and scraper were dissected under a stereoscopic microscope (Nikon, SMZ800N, Tokyo, Japan), and the surrounding muscles were removed and the samples were stored in 75% ethanol. After that, the samples were cleaned for three minutes, three times with an ultrasonic cleaner (Skymen, JP-010, Shenzhen, China), and then dehydrated in a graded ethanol series (80, 90, and 100% ethanol solution for 15 min, respectively), and dried naturally. Finally, the samples were coated with a film of gold in a sputter coater (Cressington, 108auto, London, UK), and then observed in SEM (FEI, Quattro S, Eindhoven, The Netherlands) at 15 kV.

### 2.5. Statistical Analysis

The widths of ridges in the file of 15 females and 15 males of each of the three species of longhorn beetles were measured five times for each sample using Image-J (v1.8.0). The time and frequency domains of the acoustic signals of the adult longhorn beetles were observed using MATLAB R2022b. The Kruskal–Wallis test and Mann–Whitney U test were used to test the significance of the differences in the widths of ridges in the file and the duration of the sound signal. Statistical analyses were performed with SPSS 27.0.

## 3. Results

### 3.1. Morphology of Stridulatory Organs

**Figure 1:**
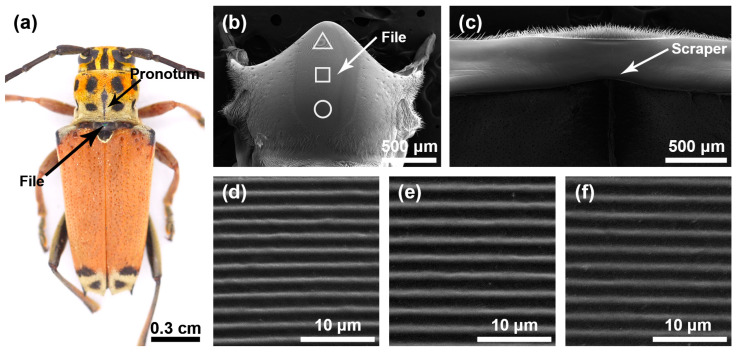
Stridulatory organs of *Glenea cantor*. (**a**) Pronotum and file of *Glenea cantor* female. (**b**) The overall structure of the file. (**c**) The overall structure of the scraper that is located on the inner surface of the pronotum. (**d**) Magnified view of the front part of file (triangle). (**e**) Magnified view of the center part of file (square). (**f**) Magnified view of the rear part of file (circular).

**Figure 2:**
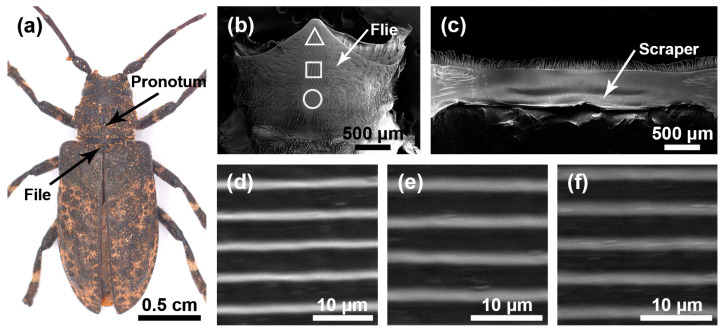
Stridulatory organs of *Moechotypa diphysis*. (**a**) Pronotum and file of *Moechotypa diphysis* female. (**b**) The overall structure of the file. (**c**) The overall structure of the scraper that is located on the inner surface of the pronotum. (**d**) Magnified view of the front part of file (triangle). (**e**) Magnified view of the center part of file (square). (**f**) Magnified view of the rear part of file (circular).

**Figure 3:**
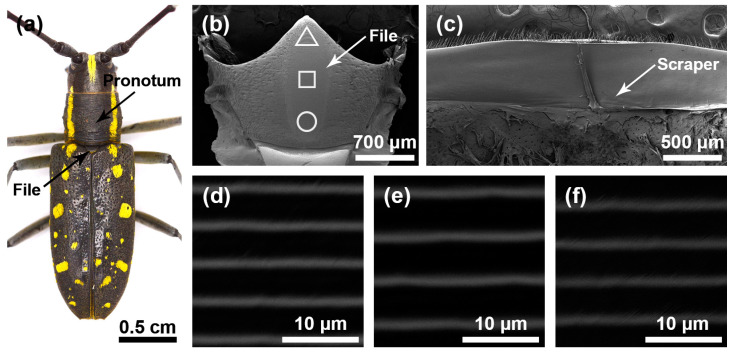
Stridulatory organs of *Psacothea hilaris*. (**a**) Pronotum and file of *Psacothea hilaris* female. (**b**) The overall structure of the file. (**c**) The overall structure of the scraper that is located on the inner surface of the pronotum. (**d**) Magnified view of the front part of file (triangle). (**e**) Magnified view of the center part of file (square). (**f**) Magnified view of the rear part of file (circular).

The stridulatory organs of the three species of longhorn beetles were the same, consisting of a file and a scraper, with the file located in the central area of the mesoscutum and the scraper located on the inner surface of the pronotum. There were many horizontally arranged ridges distributed on the file. In the natural state, the file was covered by the pronotum (Figure 1a, Figure 2a and Figure 3a). In each species, the basic structures of the male and the female stridulatory organs were also the same except the widths of the ridges. Here, we only show the female stridulatory organs in Figure 1, Figure 2 and Figure 3.

The file shape of *G. cantor* was approximately oval (Figure 1b), with a small number of sensilla trichodea around the file. There were many slender and horizontal ridges distributed on the file (Figure 1d–f). The spacing of the transverse ridges was 1.371 ± 0.054 μm. These ridges rubbed against the scraper on the pronotum (Figure 1c) to produce sounds. The width of the ridges in areas I, II, and III of the female insects were as follows: 0.387 ± 0.071 μm, 0.481 ± 0.056 μm, 0.423 ± 0.059 μm, respectively, and there were significant differences among the three areas (*χ*^2^ = 65.509, *p* < 0.01); the widths of the ridges in areas I, II, and III of male insects were as follows: 0.281 ± 0.056 μm, 0.341 ± 0.105 μm, and 0.315 ± 0.056 μm, respectively, and there were significant differences among the three areas (*χ*^2^ = 20.118, *p* < 0.01).

The file shape of *M. diphysis* was similar to a long fusiform (Figure 2b), and there were many sensilla trichodea around the file. There were many slender and horizontal ridges distributed on the file (Figure 2d–f). The spacing of the transverse ridges was 4.346 ± 0.120 μm. These ridges rubbed against the scraper on the pronotum (Figure 2c) to produce sounds. The widths of the ridge in areas I, II, and III of female insects were as follows: 0.402 ± 0.081 μm, 0.507 ± 0.128 μm, and 0.417 ± 0.066 μm, respectively, and there were significant differences among the three areas (*χ*^2^ = 38.219, *p* < 0.01); the widths of the ridges in areas I, II, and III of male insects were as follows: 0.377 ± 0.067 μm, 0.430 ± 0.057 μm, and 0.299 ± 0.039 μm, respectively, and there were significant differences among the three areas (*χ*^2^ = 118.500, *p* < 0.001).

The file shape of *P. hilaris* was also like a long fusiform (Figure 3b), and there were many sensilla trichodea around the file. There were many slender and horizontal ridges distributed on the file (Figure 3d–f). The spacing of the transverse ridges was 4.261 ± 0.095 μm. These ridges rubbed against the scrapers on the pronotum (Figure 3c) to produce sounds. The widths of the ridge in areas I, II, and III of female insects were as follows: 0.551 ± 0.074 μm, 0.596 ± 0.070 μm, and 0.538 ± 0.053 μm, respectively, and there were significant differences among the three areas (*χ*^2^ = 24.295, *p* < 0.01); the widths of the ridges in areas I, II, and III of male insects were as follows: 0.413 ± 0.052 μm, 0.462 ± 0.071 μm, and 0.444 ± 0.066 μm, respectively, and there were significant differences among the three areas (*χ*^2^ = 18.827, *p* < 0.01).

**Figure 4:**
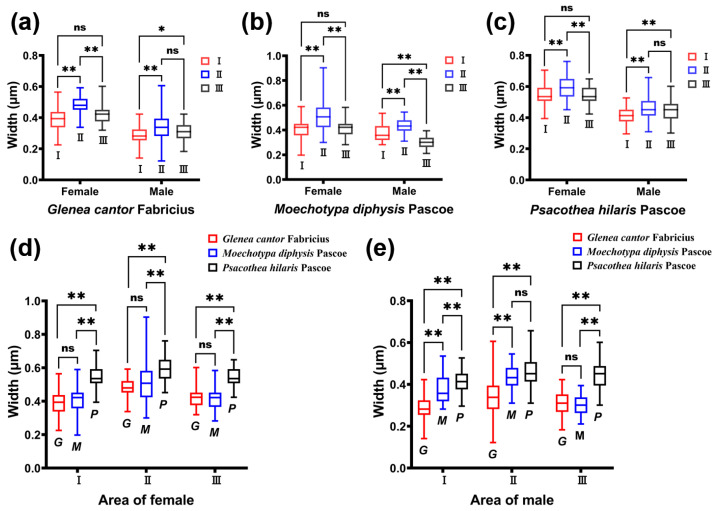
Comparison of the width of the ridges of three species of longhorn beetles. (**a**) Comparison of the width of ridges in different areas of *Glenea cantor* females and males. (**b**) Comparison of the width of ridges in different areas of *Moechotypa diphysis* females and males. (**c**) Comparison of the width of ridges in different areas of *Psacothea hilaris* females and males. (**d**) Comparison of the width of ridges in the same area in three female longhorn beetles. (**e**) Comparison of the width of ridges in the same area in three male longhorn beetles. Ns, No significant difference between sexes by the Kruskal–Wallis test. *, Significant difference between sexes at the 0.05 level as determined via the Kruskal–Wallis. **, Significant difference between sexes at the 0.01 level as determined via the Kruskal–Wallis test.

The density of the ridges of *G. cantor* was higher than that of the other two longhorn beetles, and the density of *M. diphysis* was consistent with that of *P. hilaris*. We compared the width of the ridges between males and females of the three species of longhorn beetles in the same area and found that there were significant differences in the width of the ridges between male and female longhorn beetles of the three species in the same area (Table 1). Among these three species of longhorn beetles, the width of the ridges in area II was large, and the width of the ridges of females was larger than that of males (Table 1). This phenomenon was also observed in the other two areas. In female insects, there were significant differences between area I and II, and area II and III (Figure 4a–c). However, there was no significant difference between area I and area III. In male insects, there were significant differences between area I and II, and area I and III (Figure 4a–c), but there was no significant difference between area II and III, except for *M. diphysis* (Figure 4b).

We also compared the width of the ridges in the same area among the three species of longhorn beetles. It was found that there were significant differences in the three areas in female adults (I: *χ*^2^ = 114.113, *p* < 0.001; II: *χ*^2^ = 68.631, *p* < 0.01; III: *χ*^2^ = 104.875, *p* < 0.001); there were also significant differences in the three areas in male adults (I: *χ*^2^ = 113.622, *p* < 0.001; II: *χ*^2^ = 69.254, *p* < 0.001; III: *χ*^2^ = 123.347, *p* < 0.001). In females, there were significant differences between area I and area III, and between area II and area III (Figure 4d). In males, the widths of the ridges of the three species of longhorn beetles showed significant differences in area I (Figure 4e). In area II, only the widths of the ridges of *M. diphysis* and *P. hilaris* showed no significant difference, while the others showed significant differences. In area III, only the widths of the ridges of *G. cantor* and *M. diphysis* showed no significant difference, while the others showed significant differences (Figure 4e).

### 3.2. Sound Analysis

All three species of longhorn beetles produced sounds by rubbing the file and the scraper against each other, which was like a “nodding” movement, moving the head forward and back. The sounds emitted by different longhorn beetles have certain specificities. When analyzing sound characteristics, commonly used indicators include time and frequency domains. Time domain features mainly include pulse number (PN), pulse group duration (PGD), pulse group interval (PGI), pulse duration (PD), and pulse interval (PI). Frequency domain features often include the dominant frequency (DF). The time required for a longhorn beetle to finish a complete sound is called PGD, which includes pre-pulse duration (PPD), PI, and after-pulse duration (APD).

**Figure 5:**
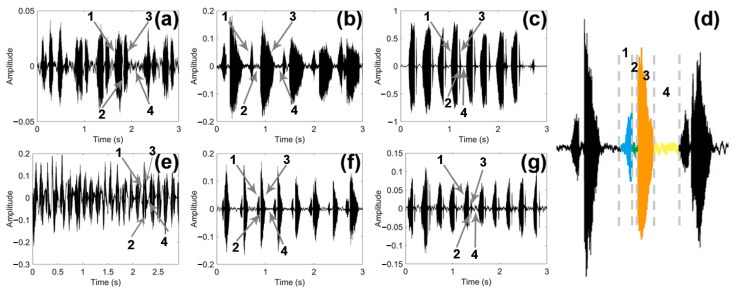
Time domain graph of the sound of three species of longhorn beetles. (**a**) Time domain graph of the sound in *Glenea cantor* females. (**b**) Time domain graph of the sound in *Moechotypa diphysis* females. (**c**) Time domain graph of the sound in *Psacothea hilaris* females. (**d**) Graph of a complete sound made by *Moechotypa diphysis* males, the blue part (1) is PPD, the green (2) part is PI, the orange part (3) is APD, and the yellow part (4) is PGI. (**e**) Time domain graph of the sound in *Glenea cantor* males. (**f**) Time domain graph of the sound in *Moechotypa diphysis* males. (**g**) Time domain graph of the sound in *Psacothea hilaris* males.

By observing and comparing the time domain graph of the sounds of the three male and female longhorn beetles (Figure 5), we found that the sounds of the three male and female longhorn beetles have certain differences. The blue part (1) was PPD, the green (2) part was PI, the orange part (3) was APD, and the yellow part (4) was PGI; the time of these four parts constituted a complete sound of the longhorn beetle (Figure 5d). These three species of longhorn beetles can complete multiple sounds in a short period of time.

We compared the PPD, PI, APD, and PGD between male and female adults of the three species of longhorn beetles (Table 2). There were significant differences in the four durations between male and female adults of *G. cantor*, no significant differences between male and female adults of *M. diphysis*, and significant differences in the three durations between male and female adults of *P. hilaris* (Table 2). Except that the APD of males of *M. diphysis* was longer than that of females and PI of males of *P. hilaris* was longer than that of females, the duration of females was longer than that of males at other types of time.

**Figure 6:**
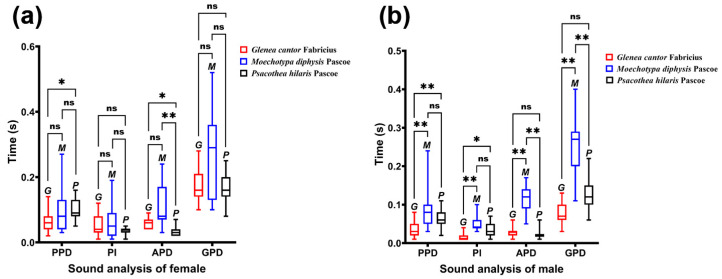
Comparison of the sounds of three female longhorn beetles. (**a**) Comparison of the sounds of three female longhorn beetles in different sections. (**b**) Comparison of the sounds of three male longhorn beetles in different sections. Ns, No significant difference between sexes by the Kruskal–Wallis test. *, Significant difference between sexes at the 0.05 level as determined via the Kruskal–Wallis. **, Significant difference between sexes at the 0.01 level as determined via the Kruskal–Wallis test.

We compared the differences in duration of different types among the three species of longhorn beetles (Figure 6). In females, only PPD and APD were significantly different among species (PPD: *χ*^2^ = 6.869, *p* = 0.032; APD: *χ*^2^ = 22.914, *p* < 0.01), and there was no significant difference in PI and GPD (PI: *χ*^2^ = 1.159, *p* = 0.560; GPD: *χ*^2^ = 4.044, *p* = 0.132). In males, the duration of each type was significantly different among species (PPD: *χ*^2^ = 17.732, *p* < 0.01; PI: *χ*^2^ = 21.728, *p* < 0.01; APD: *χ*^2^ = 30.173, *p* < 0.01; GPD: *χ*^2^ = 30.733, *p* < 0.01).

### 3.3. Sound Recognition

**Figure 7:**
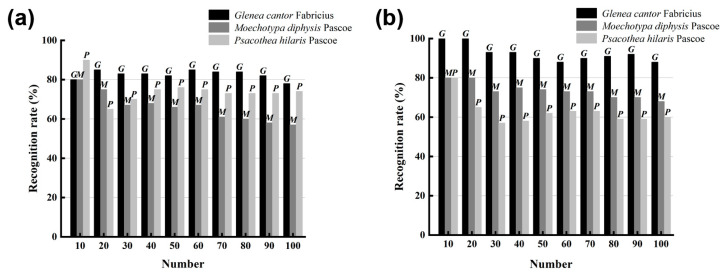
Comparison of two feature extraction methods. (**a**) Recognition of LPCC and SVM. (**b**) Recognition of MFCC and SVM.

For the recognition of the sound of longhorn beetles, LPCC and MFCC were used to extract the features of the sound of longhorn beetles, and the recognition model used was SVM. As the number of samples to be tested increased from 10 to 100, the recognition rates of the three longhorn beetle sounds were also changed (Figure 7). Using the LPCC and SVM method for recognition (Figure 7a), the average recognition rate of *G. cantor* was 82.6%, the average recognition rate of *M. diphysis* was 74.4%, and the average recognition rate of *P. hilaris* was 65.9%; using the MFCC and SVM method for recognition (Figure 7b), the average recognition rate of *G. cantor* was 92.5%, the average recognition rate of *M. diphysis* was 73.6%, and the average recognition rate of *P. hilaris* was 62.6%. Although the recognition results of *M. diphysis* and *P. hilaris* using the MFCC and SVM method were lower than those of the LPCC and SVM, the overall recognition effect of the combination of MFCC and SVM was better.

## 4. Discussion

In this paper, we studied the stridulatory organs and sound characteristics of three adult longhorn beetles of Lamiinae. All three species of longhorn beetles produce sounds relying on the friction of the thoracic stridulatory organs, which were all composed of the file on the mesoscutum and the scraper on the pronotum. This study divided the file on the mesothorax into three areas, and compared the differences between males and females in these three areas. The sounds of the three species of longhorn beetles were also collected to analyze acoustic features. The MFCC and SVM method and LPCC and SVM method were used to recognize and classify longhorn beetles, respectively, and the overall recognition effect of the combination of MFCC and SVM was better.

Many insects emit sounds relying on friction [33,34]. For example, there are differences in the number and arrangement of teeth on the stridulatory organs of stag beetles among different species, and the sound signals are composed of different numbers of pulses, which also show species-specific differences [35]. In longhorn beetles of Lamiinae, the width of the sound teeth shows significant differences among different species, and thus generate specific friction sounds [36]. The sound of *Batocera lineolata* (Hope) consists of a forward chirping sound and a backward chirping sound, with a brief pause between these two chirping sounds, and there is a difference in the duration of these two chirping sounds [37]. The stridulatory mechanism and acoustic signal of the longhorn beetles studied in this paper are like that of Lamiinae reported by other researchers [30,36,37]. The sound is produced by the friction between the scraper on the pronotum and the file on the mesoscutum. The width of the ridges and the shape of the scrape may affect the specificity of the sound produced by the beetles.

To study the sounds of insects, it is necessary to conduct acoustic analysis of the sounds emitted by insects. Acoustic analysis mainly includes the stridulatory mechanism, the components of sound, and the situation of sound signals in the time and frequency domain [38,39]. In previous studies, many researchers used the differences in the stridulatory mechanism and the components of sound to monitor and identify insects [40,41]. For larvae of longhorn beetles *Anoplophora glabripennis* (Motschulsky), *Neoplocaederus ferrugineus* (Linnaeus), and *Mallodon dasystomus* (Say), the acoustic detection methods can be used to analyze the degree of damage to the host trees [28,42,43]. The sounds of the *Hylurgus ligniperda* can be distinguished between individuals, and there is sexual dimorphism in the stridulatory organ [44]. The acoustic analysis was also conducted by Kawakita and Ichikawa [24] to investigate how to accurately identify bee species. In this paper, we not only studied the stridulatory organs of longhorn beetles, but also identified and classified their sounds. Our sound recognition methods may provide great help in solving the difficult problem in identifying longhorn beetle species.

Using machine learning (ML) methods to identify insect sounds can help people better produce and live. The buzzing of bees can be identified to better distinguish bees that are beneficial to tomato pollination and increase tomato yields; the recognition rate of MFCC combined with SVM is only 73.99% [45]. The ML method is used to identify bee sounds from bee sounds, cricket sounds, and noise. The recognition result of MFCC and SVM combined after adding standard deviation is above 90% [46]. We used MFCC and SVM to identify and classify the sounds of longhorn beetles; the best recognition result was above 90%, and the poor recognition result was above 60%. Our results also support the idea that the combination of MFCC and SVM has a good effect on insect recognition, but factors such as audio quality, feature extraction methods, and the choice of recognition model will affect the recognition results. Only by trying different methods and perfecting the improvement steps can the recognition rate be improved.

In conclusion, the subtle difference in the stridulatory organs of longhorn beetles is one of the reasons why the sounds they make are different. By taking advantage of the difference in the sounds emitted by longhorn beetles, sound recognition technology can be used to complete recognition and classification based on the sounds of longhorn beetles. Certainly, there are also many successful examples in other insects. The combination of information technology and agriculture will be one of the research hotspots in the future.

## Figures and Tables

**Table 1:** Comparison of the width of the ridges of three species of longhorn beetles between males and females.

Species	Area	Width (μm) Mean ± SD	Width (μm) Mean ± SD	z	*p*
		♀	♂		
*Glenea cantor*	I	0.387 ± 0.071 **	0.281 ± 0.056	−8.047	*p* < 0.01
	II	0.481 ± 0.056 **	0.341 ± 0.105	−8.267	*p* < 0.001
	III	0.423 ± 0.059 **	0.315 ± 0.056	−8.595	*p* < 0.001
*Moechotypa diphysis*	I	0.402 ± 0.081 **	0.377 ± 0.067	−2.600	*p* = 0.009
	II	0.507 ± 0.128 **	0.430 ± 0.057	−4.355	*p* < 0.01
	III	0.417 ± 0.066 **	0.299 ± 0.039	−9.194	*p* < 0.001
*Psacothea hilaris*	I	0.551 ± 0.074 **	0.413 ± 0.052	−9.305	*p* < 0.001
	II	0.596 ± 0.070 **	0.462 ± 0.071	−8.612	*p* < 0.001
	III	0.538 ± 0.053 **	0.444 ± 0.066	−7.808	*p* < 0.01

** Significant difference between sexes at the 0.01 level as determined via the Mann–Whitney U test.

**Table 2:** Comparison of the duration of each sound segment between males and females of three species of longhorn beetles.

Species	Time	Time(s) Mean ± SD		z	*p*
		♀	♂		
*Glenea cantor*	PPD	0.067 ± 0.031 **	0.034 ± 0.017	−3.339	*p* < 0.01
	PI	0.049 ± 0.032 **	0.017 ± 0.010	−3.364	*p* < 0.01
	APD	0.059 ± 0.016 **	0.027 ± 0.013	−4.212	*p* < 0.01
	PGD	0.177 ± 0.055 **	0.078 ± 0.029	−4.349	*p* < 0.01
*Moechotypa diphysis*	PPD	0.095 ± 0.070	0.085 ± 0.050	−0.062	*p* = 0.950
	PI	0.060 ± 0.051	0.049 ± 0.018	−0.293	*p* = 0.770
	APD	0.110 ± 0.065	0.120 ± 0.035	−1.021	*p* = 0.307
	PGD	0.265 ± 0.133	0.254 ± 0.067	−0.416	*p* = 0.678
*Psacothea hilaris*	PPD	0.099 ± 0.029 **	0.065 ± 0.026	−2.940	*p* = 0.003
	PI	0.035 ± 0.011	0.036 ± 0.017	−0.021	*p* = 0.983
	APD	0.033 ± 0.015 *	0.025 ± 0.013	−2.308	*p* = 0.021
	PGD	0.167 ± 0.043 *	0.125 ± 0.041	−2.519	*p* = 0.012

* Significant difference between sexes at the 0.05 level. ** Significant difference between sexes at the 0.01 level as determined via the Mann–Whitney U test.

## Data Availability

The data presented in this study are available on request from the corresponding author.
